# Is Health Practitioner Regulation Keeping Pace with the Changing Practitioner and Health-Care Landscape? An Australian Perspective

**DOI:** 10.3389/fpubh.2016.00091

**Published:** 2016-06-13

**Authors:** Jonathan Lee Wardle, David Sibbritt, Alex Broom, Amie Steel, Jon Adams

**Affiliations:** ^1^Faculty of Health, University of Technology Sydney, Ultimo, NSW, Australia; ^2^Faculty of Arts and Social Sciences, University of New South Wales, Sydney, NSW, Australia

**Keywords:** health professions, health practitioner regulation, primary care, regulation, consumer protection

## Abstract

Health-care delivery is undergoing significant evolution and change. Task substitution has resulted in some practitioner groups expanding their scope of practice by assuming more complex clinical roles, new practitioner groups have emerged, and consumer-driven demand has changed the way the public engage with health practitioners and the way many health-care services are delivered. Using Australia as a case study, this paper explores the issue of the hesitancy to include new professions in health professions regulation schemes. Despite the significant changes in the health-care delivery landscape, policy development in this area has remained relatively static, with active resistance to extending formal registration to new practitioner groups. Ignoring the issue of new practitioner groups in regulatory schemes is unacceptable from a public health perspective and runs against the key public protection objectives of health practitioner regulation. Development of pathways for the entry of new health practitioner groups into regulatory schemes must be developed as a matter of priority.

## Introduction

Historically, a major issue in protecting the public from the actions of unscrupulous practitioners was the fact that many practitioners existed outside the available regulatory models or were subject only to limited self-regulatory or pseudo-regulatory jurisdiction (e.g., accreditation by third parties, such as hospitals or insurers). As governments pursued neoliberal regulatory policies and became increasingly hesitant to register new health practitioner groups, the impact of unregistered health practitioners has increased, both in terms of new professions and increased scope for existing professions. For example, it is estimated that over 200,000 unregistered health practitioners provide health services in Australia (1). However, as such practitioners often exist outside formal organizational structures, it is difficult to estimate the true size of the sector. Using Australia as a case study, this article examines whether health practitioner regulation is keeping pace with the changing health-care landscape.

## Discussion

### The Development of the Australian National Registration and Accreditation Scheme

In 2006, the Council of Australian Governments decided to coordinate State and Territory-based statutory regulation initiatives and establish a single National Registration and Accreditation Scheme (the National Scheme) (2). This was originally for 10 nationally regulated health practitioner groups but later extended to an additional 4 “partially regulated” (i.e., regulated in at least one State or Territory) practitioner groups. The National Scheme was established with a focus on the public protective role of practitioner regulation and aimed to achieve six key objectives: protection of public safety; facilitation of workforce mobility; facilitation of high-quality education and training; facilitation of assessment of overseas-trained health practitioners; promotion of access to health services; and development of a flexible, responsive, and sustainable workforce.

While there have been some criticisms of the National Scheme’s implementation, particularly around its focus on issues such as workforce supply over public protection (3) or issues with the practical implementation of the Scheme (4), statutory regulation of health professions is still purported to offer the highest level of protection to the public (3). However, at a time when the health workforce in Australia is undergoing significant change, health practitioner regulation in Australia has remained relatively static, the challenges of transitioning to a national system of registration notwithstanding, and has failed to keep pace with the changing developments in the Australian health workforce.

### The Growing Influence and Role of Unregistered Practitioners

Recently, government investigations have paid increasing attention to the growing number of complaints against unregistered health practitioners, highlighting the inadequacy of current legislative and regulatory tools to appropriately deal with such complaints (1). This has included mooting the national roll-out of “negative licensing” legislation (as currently existing in New South Wales and South Australia) *via* the National Scheme. This model is based around a Statutory Code of Conduct for all the health practitioners, violation of which can result in the issuance of a prohibition order (essentially banning or placing conditions upon an individual’s practice), of which non-compliance is a criminal offense.

However, analysis of the New South Wales experience of negative licensing shows that while such legislation offers some new degree of protection to the public and serves as an excellent “catch-all” safety net for previous regulatory gaps, it does not appear to fulfill the same protective role as statutory regulation of health practitioners (5). As such, it should not be viewed as a *replacement* for extension of statutory registration to new health practitioner groups, but rather as a *complementary* measure to existing and new statutory registration arrangements.

### The Changing Roles of Australian Health Practitioners

The Australian health workforce is also evolving to meet the changing needs and shifting priorities of the contemporary health system. For example, task substitution and task delegation have long been a focus of Australian health policy and health workforce planning (6). More recently, it has been proposed that health practitioners need to work toward the “top of their license,” performing health-care tasks that, while legally and technically allowable, were not previously part of their standard practice or professional training (7). High-level tasks, once restricted to medical practitioners, have been promoted by Australian governments for task substitution, including reporting pathology and X-rays (scientists and medical imaging technologists replacing pathologists and radiologists), administering anesthesia (nurse anesthetists replacing anesthetists), and laryngoscopy (speech pathologists replacing ENT surgeons) (8).

As the Australian health-care system becomes increasingly complex, consumer-driven, and patient-centered, many practitioner groups have expanded their scope of practice into new areas. For example, the increasing number of dietitians entering unsupervised private practice in one-on-one clinical settings (9) may negate assumptions that dietitians, for example, do not require statutory regulation, as dietetic practice was focused on hospital practice under the supervision of registered health practitioners (employment and supervision being held to a form of a regulation suitable for some unregistered practitioners) (10). Increasing specialization has resulted not only in long-established practitioner groups, such as psychiatry and psychology, entering new fields of practice but also in the emergence of newer mental health disciplines practicing (and increasingly integrated) in practices once monopolized by psychology and psychiatry, some of whom may themselves fulfill the current criteria for future inclusion in a regulatory scheme (11).

As the Australian health system is encouraged to become more multidisciplinary, new health disciplines may develop, which may be promoted into an established role before they are fully incorporated into statutory regulatory arrangements (such as physician assistants) (12). Work previously performed by highly trained and regulated workforces is increasingly replaced by lower cost unregistered providers (such as the increasing utilization of carers and nursing assistants in areas of practice previously restricted to registered and enrolled nurses) (13).

One of the more contentious and controversial changes in the contemporary Australian health-care landscape is the increasing role of complementary and alternative medicine (CAM) practitioners. CAM practitioners may now account for half the health practitioner numbers, providing primary point-of-care service in some areas of Australia (14) as well as offering half the total health consultations and half of all out-of-pocket health spend in Australia (15). Much of the discussion, policy development, and legislative initiative surrounding unregistered health practitioners in Australia have focused on the considerable growth in utilization of CAM practitioners in the past two decades (5, 16, 17).

Inclusion of CAM practitioners in statutory registration schemes remains controversial, with detractors positing that registration grants CAM practitioners an unwarranted or undeserved legitimacy (18). Part of this controversy may come from the extraordinary heterogeneity of CAM, which is defined by exclusion (from conventional medicine) rather than any set professional, practice, or competency criteria. Heterogeneity *within* some CAM practitioner groups may be the result of their unregulated nature, for example, the recent review of evidence for natural therapies conducted by the National Health and Medical Research Council found evidence suggesting naturopathic treatment was effective for several chronic conditions, it argued that the lack of regulation of naturopaths in Australia – and the resultant variability in training and practice standards – meant that there were difficulties in translating this evidence to the Australian setting (19). Where CAM practitioners have been included in statutory regulation schemes in Australia, empirical analysis has demonstrated a public benefit, as observed in the case of Chinese medicine (20). Moreover, debate on whether specific practitioner groups “deserve” or “warrant” registration may no longer be relevant. Arguably, as health care becomes increasingly consumer-driven, the debate as to whether regulation “legitimizes professions” is increasingly moot, with lack of regulatory action only to deny minimum standards and accountability in groups already perceived by the public as legitimate by virtue of their significant utilization.

### Contemporary Construction of Health Practitioner Regulation in Australia

The contemporary policy construction of professional regulation as a process of risk management and protection of public safety and efforts by governments to reduce its use as a tool for developing professional monopolies can be quite different from sociological perspectives that have often theorized statutory regulation as the state’s legitimation and protection of the regulated group’s work (21). The ideation of regulation as an essential element of professionalism has contributed to theoretical constructs of state regulation as a legitimation tool, as the process of regulation has historically been politicized by occupations for professional benefit, often with little impact on standards of care, professional integrity, or public trust (22, 23). To reduce politicization of the issue of registration of new health practitioner groups, Australian governments have taken an increasingly hesitant position on registering new professions. In 1981, the Standing Committee of the Conference of Australian Health Ministers concluded that the registration of health practitioners was granted too readily (24) and set in motion developments that ultimately led to the development of formal criteria for assessing the regulatory requirements of unregulated health occupations in 1995, based on public interest criteria, which remain in effect today as the *Intergovernmental Criteria (IGA) for Assessing the Need for Statutory Regulation of Unregulated Health Professions* (outlined in Table 1). The most recent review of the National Scheme has requested further review of these criteria, though they are unlikely to change significantly.

**Table 1:** **Intergovernmental criteria for assessing the need for statutory regulation of unregulated health professions**.

**Criterion 1**
It is appropriate for health ministers to exercise responsibility for regulating the occupation in question, or does the occupation more appropriately fall within the domain of another ministry?
**Criterion 2**
Do the activities of the occupation pose a significant risk of harm to the health and safety of the public?
**Criterion 3**
Do existing regulatory or other mechanisms fail to address health and safety issues?
**Criterion 4**
Is regulation possible to implement for the occupation in question?
**Criterion 5**
Is regulation practical to implement for the occupation in question?
**Criterion 6**
Do the benefits to the public of regulation clearly outweigh the potential negative impact of such regulation?

Development of formal criteria for health practitioner regulation was complemented by an initial attempt at national standardization of legislation, such as the *Mutual Recognition Act 1992*, which resulted in the removal of registration of some practitioner groups [speech pathologists and social workers in the Northern Territory under the *Health Practitioners and Allied Professions Act 1985* (NT) and dietitians in Victoria under the *Dietitians Act 1981* (Vic)] not then registered across multiple jurisdictions (5). Regulatory arrangements liberalized further in the 1990s as part of the Competition Review, which recommended removal of anti-competitive provisions within health practitioner regulation, particularly the use of regulation to establish and protect professional monopolies (25).

Additionally, no formal pathway for consideration of new practitioner groups in the National Scheme (beyond “application for consideration”). This results in any expansion of the scheme occurring in an *ad hoc*, non-systematic manner. For example, while Victorian government work to register naturopaths and herbalists based on formal assessment against the *IGA Criteria* [(17), p. 64; Ref. (26)] was passed on to COAG with the move to a National Scheme since 2008, the only new group being considered (paramedics) was unilaterally proposed by the Western Australian health minister before such an assessment had been made (27). A formalized independent process is required to ensure such decisions are made solely based on public benefit and is not reliant on political will or support. Such decisions also need to be timely – lack of progress on inclusion of paramedics has resulted in Victoria, South Australia, and Tasmania developing independent regulatory provisions outside the National Scheme to ensure public interests are met, further eroding a truly national or systematic approach to health practitioner registration.

### The Politics of Entry of New Professions

The most recent review of the National Scheme acknowledges the potential political motivation behind some professions seeking inclusion in the National Scheme, noting “it must be remembered that the National Scheme was established to fulfil four key objectives [based around public protection and safety], not to provide status and credibility to health practitioner groups” (10). However, current mechanisms for entry encourage, rather than discourage, this perception and behavior by aspirant groups. AHMAC states that the inclusion of new groups in the National Scheme requires a “request” or “application” from the practitioner group itself for consideration (1), p. 26. Notwithstanding the fact that no formal pathway exists for such requests, this also inappropriately positions registration as a “prize” that practitioner groups need to actively attain, rather than encourage independent assessment to identify which particular practitioner groups warrant consideration for inclusion in the National Scheme based on the *IGA Criteria*. A recent review of the National Scheme even proposed the option of amending the National Scheme legislation to “recognize” professions that “provide adequate public protection through other regulatory means” (10). This approach would not only be antithetical to the objectives that health practitioner regulation is not about recognition but also offer little or no guarantee that appropriate protections would be maintained or enforced over time.

Requiring application from aspirant groups not only assumes that practitioner groups have united representation on such issues (most do not) but also allows opposition to further levels of accountability and minimum standards or fragmentation within practitioner groups to derail potential registration, even where such assessment for inclusion may be warranted. For example, while the South Australian investigation into unregistered, deregistered, and bogus practitioners specifically identified naturopathy and counseling/psychotherapy as two practitioner groups for which additional regulatory requirements beyond “negative licensing” may be required (28), historically vested interests within these professions have vociferously opposed any attempts to impose independent accountability measures (5).

## Summary

A review of the National Scheme has resulted in the Council of Australian Governments further delaying entry of new professions, under the guise of additional reviews of the criteria for registration. The changing nature of health-care provision in Australia necessitates that the issue of considering new practitioner groups in regulatory arrangements (such as the National Scheme in Australia) be made a priority. Registration of health practitioners has largely failed to keep pace with changes in the health landscape and has failed to remove the politicization of registration as an issue of public protection rather than professional legitimacy. Inclusion of new health practitioner groups into regulatory arrangements needs to be conducted independently, not reliant on professions’ applying for registered status themselves, and based solely on whether professions should be registered to protect the public, not only whether new professions “warrant” or “deserve” registration.

For example, in Australia, rather than “application” processes, independent assessment of all major currently unregistered health practitioner groups for inclusion against the *IGA Criteria* should be undertaken, which would be consistent with the objectives and guiding principles (focusing on public protection rather than professional recognition) of the National Scheme, which originally highlighted a formalized assessment stage for currently unregistered health professions (1), p. 49. While this article focuses on the Australian experience, many of the issues are also of international significance, where there can be a reluctance to grant registration for a variety of reasons unrelated to the public interest. For example, in the United Kingdom, despite statutory registration of herbalists being recommended by a Department of Health guidelines and consistent with World Health Organization recommendations (29), such action was denied by the health secretary to avoid giving the group the “full trappings of professional recognition that are applied to practitioners of orthodox health care” (30), instead moving the group to a voluntary scheme that was already known to be ineffective (5). Task substitution and the development of new professions is an issue that occurs internationally, albeit in manners that are usually unique to individual regional circumstances.

Continuing to ignore the issue of including new practitioner groups in the National Scheme or ignoring the assessments against pre-determined criteria is unacceptable from a public health perspective and runs against the key objectives of the principles of health practitioner regulation (focused as they are on public protection). If health practitioner regulation is to be truly focused on ensuring the public interest is upheld, the development of pathways for entry of new health practitioner groups into regulatory schemes in both Australia and internationally need to be developed as a matter of priority.

## Author Contributions

JW designed and carried out the evaluation and conceived of and drafted the manuscript. DS, AB, JA, and AS made substantial contributions to the design of the evaluation, collection, and interpretation of the data and critically revised the manuscript. All authors provided important intellectual content drawn from their specific academic and clinical disciplines and fields of expertise, which provided multidisciplinary insights into the article. All authors read and approved the final manuscript.

## Conflict of Interest Statement

The authors declare that the research was conducted in the absence of any commercial or financial relationships that could be construed as a potential conflict of interest.
